# Supplementary material for: Prototype of running clinical trials in an untrustworthy environment using blockchain
Source: Nat Commun. 2019 Feb 22;10:917. doi: 10.1038/s41467-019-08874-y (PMC6384889; doi:10.1038/s41467-019-08874-y)
Supplement: Supplementary file 1 — Supplementary Information [file 41467_2019_8874_MOESM1_ESM.pdf]

Supplementary Information

Wong et al.

The following document contains all of the supplementary information and data for the paper: *Prototype of Running Clinical Trials in an Untrustworthy Environment Using Blockchain*. A brief table of contents is below.

**Section**	**Page Number**
Supplementary Methods	2
Supplementary Discussion	3
Supplementary Tables	4
Supplementary Notes	5
Supplementary Note 1	5
Supplementary Note 2	12
Supplementary Note 3	19
Supplementary Note 4	23

Supplementary Methods

The blockchain in this simulation exists as multiple copies in one physical location on one machine (see README on GitHub: https://github.com/wongdaniel8/ClinicalTrials/). In reality, the blockchain will be duplicated and stored on multiple different machines, all controlled by the regulator, so that if one machine fails or is corrupted, the network data is not jeopardized. The true data and hash values will be reached by consensus of the different machines, such that each machine will cast a vote on what the hash values of particular articles of data should be. The majority vote consensus hash will be taken as the true value. When new transactions are added on the clinical network, the transactions will be appended to each blockchain on each physically separated machine. This schema will appropriately guard against the compromise or failure of individual machines, given enough machines are used in data storage and no more than half of them are compromised. When validating a blockchain, hash checks are performed chronologically from the genesis block. All elements of the block are hashed using SHA256, and this hash string is compared to the ground truth hash that is reached by consensus.

Supplementary Discussion

This scheme, if taken from the current proof of concept prototype to a fully functional service, also has the potential to meet scalability needs as more machines and more memory to these machines will be added as needed. With the advent of massive cloud storage, space availability is not an issue, and runtime is still linear in the amount of data on a trial's blockchain. Elements such as timestamp, sender and receiver identities, and hashes, all of which are small string literals, add a negligible fixed-size burden (k bytes) to the memory allocation requirements per entry. More formally, given a memory burden of n bytes for all files stored on the chain with m number of machines, the system requires $n + k$ bytes duplicated across m machines, or $m(n + k)$ memory allocation, which is bounded by O(n), and hence linearly scalable. Performance is also linear. Checking for cryptographic hash equivalence is simply a string matching test of fixed length strings returned from SHA256, which is hence bounded by a constant runtime of O(1). When constructing a hash of a file, we must inspect all elements of the data to create the hash string, which is an O(n) operation. Version controlling is fast and scalable, because checking if any two files differ simply requires checking for hash string equivalence, which is an O(1) operation. Since both memory and performance are bounded linearly, we advocate that pushing the proof of concept to full production is feasible and practical, especially given the advances in high performance computing and storage. With favorable linear bounds in place, congestion of the system through increased traffic is not an issue and performance velocity can be maintained.

Instantiating a new blockchain for a new trial is relatively simple and executed by the administrator in charge of the trialchain service. The administrator can instantiate the regulator node and all nodes in the network specified by the regulator, as is the case in private blockchains. The regulator is given full read privileges, and instantiating this super node is the same as instantiating all other nodes, just changing the specific user's privileges. Validating the user privileges and giving more or less access depending on identity is accomplished through logic in the software that is executed automatically. Full implementation of logic can be found in the GitHub repository.

Supplementary Tables

Subject Accession	Arm Accession	Verification Code
SUB73443	ARM2	c6f35fe423
SUB73452	ARM1	ed01da9a83
SUB73491	ARM3	741be0d4c0
SUB73511	ARM4	d3feabcf82

Supplementary Table 1: Subject Arm and Codes

Arm Accession	Description	Name
ARM1	Placebo omalizumab pre-treatment, placebo RIT, placebo omalizumab + placebo IT	Placebo Immunotherapy with placebo anti-IgE
ARM2	Placebo omalizumab pre-treatment, ragweed RIT, placebo omalizumab + ragweed IT	Immunotherapy with placebo anti-IgE
ARM3	Omalizumab pre-treatment, placebo RIT, omalizumab + placebo IT	Placebo Immunotherapy with anti-IgE
ARM4	Omalizumab pre-treatment, ragweed RIT, omalizumab + ragweed IT	Immunotherapy with anti-IgE

Supplementary Table 2: Arm Description

Supplementary Notes

Supplementary Note 1

The following section contains example CRF data extracted from ImmPort.

Subject: SUB73491
verification code: 741be0d4c0
Meta Information
race: Black or African American
gender: Male
age_reported: 28
arm_accession: ARM3
Blood Tests
('study_time_collected: -20 Days', 'name_reported: MCV', 'result_value_reported: 90', 'result_unit_reported: um*3')
('study_time_collected: -20 Days', 'name_reported: WBC', 'result_value_reported: 7.2', 'result_unit_reported: 10*3 cells/uL')
('study_time_collected: -20 Days', 'name_reported: MONOCYTES', 'result_value_reported: 5.4', 'result_unit_reported: %')
('study_time_collected: -20 Days', 'name_reported: MCHC', 'result_value_reported: 34', 'result_unit_reported: %')
('study_time_collected: -20 Days', 'name_reported: RBC', 'result_value_reported: 4.71', 'result_unit_reported: 10*6 cells/uL')
('study_time_collected: -20 Days', 'name_reported: BASOPHILS', 'result_value_reported: 0.2', 'result_unit_reported: %')
('study_time_collected: -20 Days', 'name_reported: MCH', 'result_value_reported: 30', 'result_unit_reported: %')
('study_time_collected: -20 Days', 'name_reported: Total IgE', 'result_value_reported: 34', 'result_unit_reported: unknown')
('study_time_collected: -20 Days', 'name_reported: HEMOGLOBIN', 'result_value_reported: 14.1', 'result_unit_reported: g/dL')
('study_time_collected: -20 Days', 'name_reported: SEGMENTED NEUTROPHILS', 'result_value_reported: 52.7', 'result_unit_reported: %')
('study_time_collected: -20 Days', 'name_reported: LYMPHOCYTES', 'result_value_reported: 37.1', 'result_unit_reported: %')
('study_time_collected: -20 Days', 'name_reported: HEMATOCRIT', 'result_value_reported: 42', 'result_unit_reported: %')
('study_time_collected: -20 Days', 'name_reported: LARGE UNCLASSIFIED CELLS', 'result_value_reported: 3.4', 'result_unit_reported: NULL')
('study_time_collected: -20 Days', 'name_reported: EOSINOPHILS', 'result_value_reported: 1.7', 'result_unit_reported: %')
('study_time_collected: -20 Days', 'name_reported: PLATELET COUNT', 'result_value_reported: 194', 'result_unit_reported: 10*3 cells/uL')
('study_time_collected: 0 Days', 'name_reported: EOSINOPHILS', 'result_value_reported: 4.6', 'result_unit_reported: %')
('study_time_collected: 0 Days', 'name_reported: RBC', 'result_value_reported: 4.45', 'result_unit_reported: 10*6 cells/uL')

('study_time_collected: 0 Days', 'name_reported: PLATELET COUNT', 'result_value_reported: 223', 'result_unit_reported: 10*3 cells/uL')

('study_time_collected: 0 Days', 'name_reported: HEMATOCRIT', 'result_value_reported: 41', 'result_unit_reported: %')

('study_time_collected: 0 Days', 'name_reported: MCV', 'result_value_reported: 92', 'result_unit_reported: um*3')

('study_time_collected: 0 Days', 'name_reported: MCHC', 'result_value_reported: 33', 'result_unit_reported: %')

('study_time_collected: 0 Days', 'name_reported: BASOPHILS', 'result_value_reported: 0.4', 'result_unit_reported: %')

('study_time_collected: 0 Days', 'name_reported: HEMOGLOBIN', 'result_value_reported: 13.5', 'result_unit_reported: g/dL')

('study_time_collected: 0 Days', 'name_reported: MCH', 'result_value_reported: 30', 'result_unit_reported: %')

('study_time_collected: 0 Days', 'name_reported: MONOCYTES', 'result_value_reported: 6.6', 'result_unit_reported: %')

('study_time_collected: 0 Days', 'name_reported: LYMPHOCYTES', 'result_value_reported: 41.2', 'result_unit_reported: %')

('study_time_collected: 0 Days', 'name_reported: WBC', 'result_value_reported: 5.9', 'result_unit_reported: 10*3 cells/uL')

('study_time_collected: 0 Days', 'name_reported: BANDS', 'result_value_reported: 0', 'result_unit_reported: %')

('study_time_collected: 0 Days', 'name_reported: SEGMENTED NEUTROPHILS', 'result_value_reported: 44.4', 'result_unit_reported: %')

('study_time_collected: 0 Days', 'name_reported: LARGE UNCLASSIFIED CELLS', 'result_value_reported: 2.8', 'result_unit_reported: NULL')

('study_time_collected: 119 Days', 'name_reported: LYMPHOCYTES', 'result_value_reported: 38.1', 'result_unit_reported: %')

('study_time_collected: 119 Days', 'name_reported: WBC', 'result_value_reported: 5.4', 'result_unit_reported: 10*3 cells/uL')

('study_time_collected: 119 Days', 'name_reported: RBC', 'result_value_reported: 4.55', 'result_unit_reported: 10*6 cells/uL')

('study_time_collected: 119 Days', 'name_reported: MCHC', 'result_value_reported: 34', 'result_unit_reported: %')

('study_time_collected: 119 Days', 'name_reported: MCV', 'result_value_reported: 88', 'result_unit_reported: um*3')

('study_time_collected: 119 Days', 'name_reported: PLATELET COUNT', 'result_value_reported: 189', 'result_unit_reported: 10*3 cells/uL')

('study_time_collected: 119 Days', 'name_reported: SEGMENTED NEUTROPHILS', 'result_value_reported: 50.2', 'result_unit_reported: %')

('study_time_collected: 119 Days', 'name_reported: MONOCYTES', 'result_value_reported: 6.2', 'result_unit_reported: %')

('study_time_collected: 119 Days', 'name_reported: HEMOGLOBIN', 'result_value_reported: 13.7', 'result_unit_reported: g/dL')

('study_time_collected: 119 Days', 'name_reported: EOSINOPHILS', 'result_value_reported: 2.4', 'result_unit_reported: %')

('study_time_collected: 119 Days', 'name_reported: LARGE UNCLASSIFIED CELLS', 'result_value_reported: 2.6', 'result_unit_reported: NULL')
('study_time_collected: 119 Days', 'name_reported: MCH', 'result_value_reported: 30', 'result_unit_reported: %')
('study_time_collected: 119 Days', 'name_reported: BASOPHILS', 'result_value_reported: 0.5', 'result_unit_reported: %')
('study_time_collected: 119 Days', 'name_reported: HEMATOCRIT', 'result_value_reported: 40', 'result_unit_reported: %')
('study_time_collected: 147 Days', 'name_reported: MCH', 'result_value_reported: 30', 'result_unit_reported: %')
('study_time_collected: 147 Days', 'name_reported: HEMOGLOBIN', 'result_value_reported: 13.7', 'result_unit_reported: g/dL')
('study_time_collected: 147 Days', 'name_reported: HEMATOCRIT', 'result_value_reported: 41', 'result_unit_reported: %')
('study_time_collected: 147 Days', 'name_reported: MCV', 'result_value_reported: 90', 'result_unit_reported: um*3')
('study_time_collected: 147 Days', 'name_reported: EOSINOPHILS', 'result_value_reported: 2.6', 'result_unit_reported: %')
('study_time_collected: 147 Days', 'name_reported: LARGE UNCLASSIFIED CELLS', 'result_value_reported: 2.4', 'result_unit_reported: NULL')
('study_time_collected: 147 Days', 'name_reported: SEGMENTED NEUTROPHILS', 'result_value_reported: 54.2', 'result_unit_reported: %')
('study_time_collected: 147 Days', 'name_reported: MONOCYTES', 'result_value_reported: 6.5', 'result_unit_reported: %')
('study_time_collected: 147 Days', 'name_reported: RBC', 'result_value_reported: 4.51', 'result_unit_reported: 10*6 cells/uL')
('study_time_collected: 147 Days', 'name_reported: PLATELET COUNT', 'result_value_reported: 203', 'result_unit_reported: 10*3 cells/uL')
('study_time_collected: 147 Days', 'name_reported: WBC', 'result_value_reported: 6.9', 'result_unit_reported: 10*3 cells/uL')
('study_time_collected: 147 Days', 'name_reported: LYMPHOCYTES', 'result_value_reported: 34', 'result_unit_reported: %')
('study_time_collected: 147 Days', 'name_reported: MCHC', 'result_value_reported: 34', 'result_unit_reported: %')
('study_time_collected: 147 Days', 'name_reported: BASOPHILS', 'result_value_reported: 0.3', 'result_unit_reported: %')
('study_time_collected: 160 Days', 'name_reported: RBC', 'result_value_reported: 4.29', 'result_unit_reported: 10*6 cells/uL')
('study_time_collected: 160 Days', 'name_reported: MONOCYTES', 'result_value_reported: 7.3', 'result_unit_reported: %')
('study_time_collected: 160 Days', 'name_reported: HEMATOCRIT', 'result_value_reported: 39', 'result_unit_reported: %')
('study_time_collected: 160 Days', 'name_reported: MCHC', 'result_value_reported: 34', 'result_unit_reported: %')
('study_time_collected: 160 Days', 'name_reported: LYMPHOCYTES', 'result_value_reported: 37', 'result_unit_reported: %')

('study_time_collected: 160 Days', 'name_reported: MCH', 'result_value_reported: 31', 'result_unit_reported: %')
('study_time_collected: 160 Days', 'name_reported: HEMOGLOBIN', 'result_value_reported: 13.3', 'result_unit_reported: g/dL')
('study_time_collected: 160 Days', 'name_reported: BASOPHILS', 'result_value_reported: 0.3', 'result_unit_reported: %')
('study_time_collected: 160 Days', 'name_reported: EOSINOPHILS', 'result_value_reported: 2.8', 'result_unit_reported: %')
('study_time_collected: 160 Days', 'name_reported: LARGE UNCLASSIFIED CELLS', 'result_value_reported: 2.9', 'result_unit_reported: NULL')
('study_time_collected: 160 Days', 'name_reported: WBC', 'result_value_reported: 4.7', 'result_unit_reported: 10*3 cells/uL')
('study_time_collected: 160 Days', 'name_reported: PLATELET COUNT', 'result_value_reported: 198', 'result_unit_reported: 10*3 cells/uL')
('study_time_collected: 160 Days', 'name_reported: MCV', 'result_value_reported: 90', 'result_unit_reported: um*3')
('study_time_collected: 160 Days', 'name_reported: SEGMENTED NEUTROPHILS', 'result_value_reported: 49.7', 'result_unit_reported: %')
('study_time_collected: 28 Days', 'name_reported: WBC', 'result_value_reported: 6.2', 'result_unit_reported: 10*3 cells/uL')
('study_time_collected: 28 Days', 'name_reported: BASOPHILS', 'result_value_reported: 0', 'result_unit_reported: %')
('study_time_collected: 28 Days', 'name_reported: BANDS', 'result_value_reported: 4', 'result_unit_reported: %')
('study_time_collected: 28 Days', 'name_reported: PLATELET COUNT', 'result_value_reported: 235', 'result_unit_reported: 10*3 cells/uL')
('study_time_collected: 28 Days', 'name_reported: LYMPHOCYTES', 'result_value_reported: 49', 'result_unit_reported: %')
('study_time_collected: 28 Days', 'name_reported: MCHC', 'result_value_reported: 33', 'result_unit_reported: %')
('study_time_collected: 28 Days', 'name_reported: EOSINOPHILS', 'result_value_reported: 0', 'result_unit_reported: %')
('study_time_collected: 28 Days', 'name_reported: SEGMENTED NEUTROPHILS', 'result_value_reported: 43', 'result_unit_reported: %')
('study_time_collected: 28 Days', 'name_reported: HEMATOCRIT', 'result_value_reported: 43', 'result_unit_reported: %')
('study_time_collected: 28 Days', 'name_reported: MCV', 'result_value_reported: 91', 'result_unit_reported: um*3')
('study_time_collected: 28 Days', 'name_reported: RBC', 'result_value_reported: 4.68', 'result_unit_reported: 10*6 cells/uL')
('study_time_collected: 28 Days', 'name_reported: MCH', 'result_value_reported: 30', 'result_unit_reported: %')
('study_time_collected: 28 Days', 'name_reported: HEMOGLOBIN', 'result_value_reported: 14.2', 'result_unit_reported: g/dL')
('study_time_collected: 28 Days', 'name_reported: MONOCYTES', 'result_value_reported: 4', 'result_unit_reported: %')

('study_time_collected: 369 Days', 'name_reported: BASOPHILS', 'result_value_reported: 0.3', 'result_unit_reported: %')

('study_time_collected: 369 Days', 'name_reported: SEGMENTED NEUTROPHILS', 'result_value_reported: 47.7', 'result_unit_reported: %')

('study_time_collected: 369 Days', 'name_reported: LYMPHOCYTES', 'result_value_reported: 39.5', 'result_unit_reported: %')

('study_time_collected: 369 Days', 'name_reported: EOSINOPHILS', 'result_value_reported: 2.3', 'result_unit_reported: %')

('study_time_collected: 369 Days', 'name_reported: HEMOGLOBIN', 'result_value_reported: 14.4', 'result_unit_reported: g/dL')

('study_time_collected: 369 Days', 'name_reported: LARGE UNCLASSIFIED CELLS', 'result_value_reported: 2.8', 'result_unit_reported: NULL')

('study_time_collected: 369 Days', 'name_reported: HEMATOCRIT', 'result_value_reported: 42', 'result_unit_reported: %')

('study_time_collected: 369 Days', 'name_reported: MCH', 'result_value_reported: 31', 'result_unit_reported: %')

('study_time_collected: 369 Days', 'name_reported: MONOCYTES', 'result_value_reported: 7.4', 'result_unit_reported: %')

('study_time_collected: 369 Days', 'name_reported: MCV', 'result_value_reported: 91', 'result_unit_reported: um*3')

('study_time_collected: 369 Days', 'name_reported: MCHC', 'result_value_reported: 34', 'result_unit_reported: %')

('study_time_collected: 369 Days', 'name_reported: RBC', 'result_value_reported: 4.62', 'result_unit_reported: 10*6 cells/uL')

('study_time_collected: 369 Days', 'name_reported: WBC', 'result_value_reported: 5.2', 'result_unit_reported: 10*3 cells/uL')

('study_time_collected: 369 Days', 'name_reported: PLATELET COUNT', 'result_value_reported: 179', 'result_unit_reported: 10*3 cells/uL')

('study_time_collected: 56 Days', 'name_reported: BANDS', 'result_value_reported: 1', 'result_unit_reported: %')

('study_time_collected: 56 Days', 'name_reported: MCV', 'result_value_reported: 90', 'result_unit_reported: um*3')

('study_time_collected: 56 Days', 'name_reported: HEMATOCRIT', 'result_value_reported: 43', 'result_unit_reported: %')

('study_time_collected: 56 Days', 'name_reported: MONOCYTES', 'result_value_reported: 9', 'result_unit_reported: %')

('study_time_collected: 56 Days', 'name_reported: HEMOGLOBIN', 'result_value_reported: 14.5', 'result_unit_reported: g/dL')

('study_time_collected: 56 Days', 'name_reported: BASOPHILS', 'result_value_reported: 0', 'result_unit_reported: %')

('study_time_collected: 56 Days', 'name_reported: MCHC', 'result_value_reported: 34', 'result_unit_reported: %')

('study_time_collected: 56 Days', 'name_reported: RBC', 'result_value_reported: 4.74', 'result_unit_reported: 10*6 cells/uL')

('study_time_collected: 56 Days', 'name_reported: MCH', 'result_value_reported: 31', 'result_unit_reported: %')

('study_time_collected: 56 Days', 'name_reported: LYMPHOCYTES', 'result_value_reported: 51', 'result_unit_reported: %')

('study_time_collected: 56 Days', 'name_reported: PLATELET COUNT', 'result_value_reported: 221', 'result_unit_reported: 10*3 cells/uL')

('study_time_collected: 56 Days', 'name_reported: WBC', 'result_value_reported: 6.7', 'result_unit_reported: 10*3 cells/uL')

('study_time_collected: 56 Days', 'name_reported: SEGMENTED NEUTROPHILS', 'result_value_reported: 39', 'result_unit_reported: %')

('study_time_collected: 56 Days', 'name_reported: EOSINOPHILS', 'result_value_reported: 0', 'result_unit_reported: %')

('study_time_collected: 91 Days', 'name_reported: BASOPHILS', 'result_value_reported: 0.3', 'result_unit_reported: %')

('study_time_collected: 91 Days', 'name_reported: LYMPHOCYTES', 'result_value_reported: 30.3', 'result_unit_reported: %')

('study_time_collected: 91 Days', 'name_reported: PLATELET COUNT', 'result_value_reported: 199', 'result_unit_reported: 10*3 cells/uL')

('study_time_collected: 91 Days', 'name_reported: MONOCYTES', 'result_value_reported: 5.5', 'result_unit_reported: %')

('study_time_collected: 91 Days', 'name_reported: EOSINOPHILS', 'result_value_reported: 1.9', 'result_unit_reported: %')

('study_time_collected: 91 Days', 'name_reported: SEGMENTED NEUTROPHILS', 'result_value_reported: 59.6', 'result_unit_reported: %')

('study_time_collected: 91 Days', 'name_reported: MCHC', 'result_value_reported: 35', 'result_unit_reported: %')

('study_time_collected: 91 Days', 'name_reported: HEMOGLOBIN', 'result_value_reported: 13.8', 'result_unit_reported: g/dL')

('study_time_collected: 91 Days', 'name_reported: LARGE UNCLASSIFIED CELLS', 'result_value_reported: 2.4', 'result_unit_reported: NULL')

('study_time_collected: 91 Days', 'name_reported: RBC', 'result_value_reported: 4.53', 'result_unit_reported: 10*6 cells/uL')

('study_time_collected: 91 Days', 'name_reported: MCH', 'result_value_reported: 30', 'result_unit_reported: %')

('study_time_collected: 91 Days', 'name_reported: MCV', 'result_value_reported: 88', 'result_unit_reported: um*3')

('study_time_collected: 91 Days', 'name_reported: HEMATOCRIT', 'result_value_reported: 40', 'result_unit_reported: %')

('study_time_collected: 91 Days', 'name_reported: WBC', 'result_value_reported: 6.8', 'result_unit_reported: 10*3 cells/uL')

Assay Measurements

('study_time_collected: -8', 'analyte: IgE-a Amb a', 'value_reported: 28.3', 'unit_reported: U/ml')

('study_time_collected: -8', 'analyte: IgG-a Amb a', 'value_reported: 43', 'unit_reported: U/ml')

('study_time_collected: -8', 'analyte: IgE-a Ragweed', 'value_reported: 1.71', 'unit_reported: kIUa/ml')

('study_time_collected: -8', 'analyte: Free IgE Concentration', 'value_reported: 89.92', 'unit_reported: ng/ml')
('study_time_collected: -8', 'analyte: IgG-a Ragweed', 'value_reported: 100', 'unit_reported: ng/ml')
('study_time_collected: 105', 'analyte: IgG-a Amb a', 'value_reported: 511.5', 'unit_reported: U/ml')
('study_time_collected: 105', 'analyte: IgE-a Amb a', 'value_reported: 3340', 'unit_reported: U/ml')
('study_time_collected: 105', 'analyte: IgE-a Ragweed', 'value_reported: 71.1', 'unit_reported: kIUa/ml')
('study_time_collected: 105', 'analyte: Free IgE Concentration', 'value_reported: 17.376', 'unit_reported: ng/ml')
('study_time_collected: 105', 'analyte: IgG-a Ragweed', 'value_reported: 385', 'unit_reported: ng/ml')
('study_time_collected: 202', 'analyte: Free IgE Concentration', 'value_reported: 34.303', 'unit_reported: ng/ml')
('study_time_collected: 202', 'analyte: IgE-a Ragweed', 'value_reported: 45.6', 'unit_reported: kIUa/ml')
('study_time_collected: 202', 'analyte: IgG-a Ragweed', 'value_reported: 377', 'unit_reported: ng/ml')
('study_time_collected: 202', 'analyte: IgG-a Amb a', 'value_reported: 398', 'unit_reported: U/ml')
('study_time_collected: 202', 'analyte: IgE-a Amb a', 'value_reported: 2195', 'unit_reported: U/ml')

Adverse Reactions

Muscle strain
Injection site swelling
Sinus headache
Insomnia
Nasal congestion
Sinus pain
Sneezing
Throat irritation
Dyspnoea exertional
Blood pressure decreased
Sinus congestion
Myalgia
Abrasion NOS
Tendon injury
Arthralgia
Skin laceration
Influenza

Supplementary Note 2

Subject: SUB73511

verification code: d3feabcf82

Meta Information

race: White

gender: Male

age_reported: 45

arm_accession: ARM4

Blood Tests

('study_time_collected: -15 Days', 'name_reported: RBC', 'result_value_reported: 5.45', 'result_unit_reported: 10*6 cells/uL')

('study_time_collected: -15 Days', 'name_reported: HEMATOCRIT', 'result_value_reported: 47', 'result_unit_reported: %')

('study_time_collected: -15 Days', 'name_reported: PLATELET COUNT', 'result_value_reported: 271', 'result_unit_reported: 10*3 cells/uL')

('study_time_collected: -15 Days', 'name_reported: WBC', 'result_value_reported: 6.7', 'result_unit_reported: 10*3 cells/uL')

('study_time_collected: -15 Days', 'name_reported: HEMOGLOBIN', 'result_value_reported: 15.8', 'result_unit_reported: g/dL')

('study_time_collected: -15 Days', 'name_reported: MCV', 'result_value_reported: 86', 'result_unit_reported: um*3')

('study_time_collected: -15 Days', 'name_reported: Total IgE', 'result_value_reported: 58', 'result_unit_reported: unknown')

('study_time_collected: -15 Days', 'name_reported: MCHC', 'result_value_reported: 34', 'result_unit_reported: %')

('study_time_collected: -15 Days', 'name_reported: MCH', 'result_value_reported: 29', 'result_unit_reported: %')

('study_time_collected: 0 Days', 'name_reported: LARGE UNCLASSIFIED CELLS', 'result_value_reported: 2.2', 'result_unit_reported: NULL')

('study_time_collected: 0 Days', 'name_reported: MONOCYTES', 'result_value_reported: 4.5', 'result_unit_reported: %')

('study_time_collected: 0 Days', 'name_reported: HEMOGLOBIN', 'result_value_reported: 14.8', 'result_unit_reported: g/dL')

('study_time_collected: 0 Days', 'name_reported: MCV', 'result_value_reported: 87', 'result_unit_reported: um*3')

('study_time_collected: 0 Days', 'name_reported: EOSINOPHILS', 'result_value_reported: 1.9', 'result_unit_reported: %')

('study_time_collected: 0 Days', 'name_reported: SEGMENTED NEUTROPHILS', 'result_value_reported: 56.3', 'result_unit_reported: %')

('study_time_collected: 0 Days', 'name_reported: HEMATOCRIT', 'result_value_reported: 44', 'result_unit_reported: %')

('study_time_collected: 0 Days', 'name_reported: WBC', 'result_value_reported: 6.8', 'result_unit_reported: 10*3 cells/uL')

('study_time_collected: 0 Days', 'name_reported: BASOPHILS', 'result_value_reported: 0.6', 'result_unit_reported: %')

('study_time_collected: 0 Days', 'name_reported: MCH', 'result_value_reported: 29', 'result_unit_reported: %')

('study_time_collected: 0 Days', 'name_reported: LYMPHOCYTES', 'result_value_reported: 34.5', 'result_unit_reported: %')

('study_time_collected: 0 Days', 'name_reported: MCHC', 'result_value_reported: 34', 'result_unit_reported: %')

('study_time_collected: 0 Days', 'name_reported: RBC', 'result_value_reported: 5.09', 'result_unit_reported: 10*6 cells/uL')

('study_time_collected: 0 Days', 'name_reported: PLATELET COUNT', 'result_value_reported: 295', 'result_unit_reported: 10*3 cells/uL')

('study_time_collected: 114 Days', 'name_reported: LARGE UNCLASSIFIED CELLS', 'result_value_reported: 2.4', 'result_unit_reported: NULL')

('study_time_collected: 114 Days', 'name_reported: PLATELET COUNT', 'result_value_reported: 278', 'result_unit_reported: 10*3 cells/uL')

('study_time_collected: 114 Days', 'name_reported: HEMATOCRIT', 'result_value_reported: 46', 'result_unit_reported: %')

('study_time_collected: 114 Days', 'name_reported: BASOPHILS', 'result_value_reported: 0.6', 'result_unit_reported: %')

('study_time_collected: 114 Days', 'name_reported: SEGMENTED NEUTROPHILS', 'result_value_reported: 57.3', 'result_unit_reported: %')

('study_time_collected: 114 Days', 'name_reported: EOSINOPHILS', 'result_value_reported: 2', 'result_unit_reported: %')

('study_time_collected: 114 Days', 'name_reported: LYMPHOCYTES', 'result_value_reported: 32.5', 'result_unit_reported: %')

('study_time_collected: 114 Days', 'name_reported: MCH', 'result_value_reported: 29', 'result_unit_reported: %')

('study_time_collected: 114 Days', 'name_reported: MCHC', 'result_value_reported: 34', 'result_unit_reported: %')

('study_time_collected: 114 Days', 'name_reported: WBC', 'result_value_reported: 7.3', 'result_unit_reported: 10*3 cells/uL')

('study_time_collected: 114 Days', 'name_reported: MONOCYTES', 'result_value_reported: 5.2', 'result_unit_reported: %')

('study_time_collected: 114 Days', 'name_reported: HEMOGLOBIN', 'result_value_reported: 15.5', 'result_unit_reported: g/dL')

('study_time_collected: 114 Days', 'name_reported: RBC', 'result_value_reported: 5.45', 'result_unit_reported: 10*6 cells/uL')

('study_time_collected: 114 Days', 'name_reported: MCV', 'result_value_reported: 85', 'result_unit_reported: um*3')

('study_time_collected: 140 Days', 'name_reported: MCV', 'result_value_reported: 84', 'result_unit_reported: um*3')

('study_time_collected: 140 Days', 'name_reported: HEMOGLOBIN', 'result_value_reported: 15.1', 'result_unit_reported: g/dL')

('study_time_collected: 140 Days', 'name_reported: LARGE UNCLASSIFIED CELLS', 'result_value_reported: 0.9', 'result_unit_reported: NULL')

('study_time_collected: 140 Days', 'name_reported: BASOPHILS', 'result_value_reported: 0.4', 'result_unit_reported: %')

('study_time_collected: 140 Days', 'name_reported: MONOCYTES', 'result_value_reported: 3.5', 'result_unit_reported: %')
('study_time_collected: 140 Days', 'name_reported: WBC', 'result_value_reported: 12.7', 'result_unit_reported: 10*3 cells/uL')
('study_time_collected: 140 Days', 'name_reported: MCH', 'result_value_reported: 29', 'result_unit_reported: %')
('study_time_collected: 140 Days', 'name_reported: EOSINOPHILS', 'result_value_reported: 0.5', 'result_unit_reported: %')
('study_time_collected: 140 Days', 'name_reported: MCHC', 'result_value_reported: 34', 'result_unit_reported: %')
('study_time_collected: 140 Days', 'name_reported: SEGMENTED NEUTROPHILS', 'result_value_reported: 77.8', 'result_unit_reported: %')
('study_time_collected: 140 Days', 'name_reported: HEMATOCRIT', 'result_value_reported: 44', 'result_unit_reported: %')
('study_time_collected: 140 Days', 'name_reported: PLATELET COUNT', 'result_value_reported: 275', 'result_unit_reported: 10*3 cells/uL')
('study_time_collected: 140 Days', 'name_reported: LYMPHOCYTES', 'result_value_reported: 16.9', 'result_unit_reported: %')
('study_time_collected: 140 Days', 'name_reported: RBC', 'result_value_reported: 5.26', 'result_unit_reported: 10*6 cells/uL')
('study_time_collected: 155 Days', 'name_reported: MCV', 'result_value_reported: 84', 'result_unit_reported: um*3')
('study_time_collected: 155 Days', 'name_reported: MONOCYTES', 'result_value_reported: 4.8', 'result_unit_reported: %')
('study_time_collected: 155 Days', 'name_reported: HEMATOCRIT', 'result_value_reported: 44', 'result_unit_reported: %')
('study_time_collected: 155 Days', 'name_reported: PLATELET COUNT', 'result_value_reported: 286', 'result_unit_reported: 10*3 cells/uL')
('study_time_collected: 155 Days', 'name_reported: LYMPHOCYTES', 'result_value_reported: 33.3', 'result_unit_reported: %')
('study_time_collected: 155 Days', 'name_reported: MCH', 'result_value_reported: 30', 'result_unit_reported: %')
('study_time_collected: 155 Days', 'name_reported: LARGE UNCLASSIFIED CELLS', 'result_value_reported: 1.7', 'result_unit_reported: NULL')
('study_time_collected: 155 Days', 'name_reported: HEMOGLOBIN', 'result_value_reported: 15.7', 'result_unit_reported: g/dL')
('study_time_collected: 155 Days', 'name_reported: BASOPHILS', 'result_value_reported: 0.9', 'result_unit_reported: %')
('study_time_collected: 155 Days', 'name_reported: WBC', 'result_value_reported: 7.2', 'result_unit_reported: 10*3 cells/uL')
('study_time_collected: 155 Days', 'name_reported: EOSINOPHILS', 'result_value_reported: 2.4', 'result_unit_reported: %')
('study_time_collected: 155 Days', 'name_reported: SEGMENTED NEUTROPHILS', 'result_value_reported: 56.9', 'result_unit_reported: %')
('study_time_collected: 155 Days', 'name_reported: RBC', 'result_value_reported: 5.21', 'result_unit_reported: 10*6 cells/uL')

('study_time_collected: 155 Days', 'name_reported: MCHC', 'result_value_reported: 36', 'result_unit_reported: %')

('study_time_collected: 28 Days', 'name_reported: SEGMENTED NEUTROPHILS', 'result_value_reported: 64.3', 'result_unit_reported: %')

('study_time_collected: 28 Days', 'name_reported: HEMOGLOBIN', 'result_value_reported: 15.8', 'result_unit_reported: g/dL')

('study_time_collected: 28 Days', 'name_reported: LARGE UNCLASSIFIED CELLS', 'result_value_reported: 1.7', 'result_unit_reported: NULL')

('study_time_collected: 28 Days', 'name_reported: RBC', 'result_value_reported: 5.44', 'result_unit_reported: 10*6 cells/uL')

('study_time_collected: 28 Days', 'name_reported: MCV', 'result_value_reported: 87', 'result_unit_reported: um*3')

('study_time_collected: 28 Days', 'name_reported: BASOPHILS', 'result_value_reported: 0.7', 'result_unit_reported: %')

('study_time_collected: 28 Days', 'name_reported: MCHC', 'result_value_reported: 33', 'result_unit_reported: %')

('study_time_collected: 28 Days', 'name_reported: PLATELET COUNT', 'result_value_reported: 267', 'result_unit_reported: 10*3 cells/uL')

('study_time_collected: 28 Days', 'name_reported: WBC', 'result_value_reported: 6.5', 'result_unit_reported: 10*3 cells/uL')

('study_time_collected: 28 Days', 'name_reported: EOSINOPHILS', 'result_value_reported: 1.3', 'result_unit_reported: %')

('study_time_collected: 28 Days', 'name_reported: LYMPHOCYTES', 'result_value_reported: 29.2', 'result_unit_reported: %')

('study_time_collected: 28 Days', 'name_reported: HEMATOCRIT', 'result_value_reported: 47', 'result_unit_reported: %')

('study_time_collected: 28 Days', 'name_reported: MONOCYTES', 'result_value_reported: 3', 'result_unit_reported: %')

('study_time_collected: 28 Days', 'name_reported: MCH', 'result_value_reported: 29', 'result_unit_reported: %')

('study_time_collected: 365 Days', 'name_reported: MCHC', 'result_value_reported: 33', 'result_unit_reported: %')

('study_time_collected: 365 Days', 'name_reported: HEMOGLOBIN', 'result_value_reported: 15.1', 'result_unit_reported: g/dL')

('study_time_collected: 365 Days', 'name_reported: SEGMENTED NEUTROPHILS', 'result_value_reported: 52.8', 'result_unit_reported: %')

('study_time_collected: 365 Days', 'name_reported: LARGE UNCLASSIFIED CELLS', 'result_value_reported: 1.7', 'result_unit_reported: NULL')

('study_time_collected: 365 Days', 'name_reported: MCV', 'result_value_reported: 87', 'result_unit_reported: um*3')

('study_time_collected: 365 Days', 'name_reported: MONOCYTES', 'result_value_reported: 6.2', 'result_unit_reported: %')

('study_time_collected: 365 Days', 'name_reported: RBC', 'result_value_reported: 5.19', 'result_unit_reported: 10*6 cells/uL')

('study_time_collected: 365 Days', 'name_reported: HEMATOCRIT', 'result_value_reported: 45', 'result_unit_reported: %')

('study_time_collected: 365 Days', 'name_reported: EOSINOPHILS', 'result_value_reported: 2.7', 'result_unit_reported: %')
('study_time_collected: 365 Days', 'name_reported: LYMPHOCYTES', 'result_value_reported: 36.3', 'result_unit_reported: %')
('study_time_collected: 365 Days', 'name_reported: MCH', 'result_value_reported: 29', 'result_unit_reported: %')
('study_time_collected: 365 Days', 'name_reported: WBC', 'result_value_reported: 5.9', 'result_unit_reported: 10*3 cells/uL')
('study_time_collected: 365 Days', 'name_reported: BASOPHILS', 'result_value_reported: 0.3', 'result_unit_reported: %')
('study_time_collected: 365 Days', 'name_reported: PLATELET COUNT', 'result_value_reported: 252', 'result_unit_reported: 10*3 cells/uL')
('study_time_collected: 56 Days', 'name_reported: RBC', 'result_value_reported: 5.17', 'result_unit_reported: 10*6 cells/uL')
('study_time_collected: 56 Days', 'name_reported: SEGMENTED NEUTROPHILS', 'result_value_reported: 58.9', 'result_unit_reported: %')
('study_time_collected: 56 Days', 'name_reported: PLATELET COUNT', 'result_value_reported: 270', 'result_unit_reported: 10*3 cells/uL')
('study_time_collected: 56 Days', 'name_reported: MCH', 'result_value_reported: 30', 'result_unit_reported: %')
('study_time_collected: 56 Days', 'name_reported: BASOPHILS', 'result_value_reported: 0.7', 'result_unit_reported: %')
('study_time_collected: 56 Days', 'name_reported: LARGE UNCLASSIFIED CELLS', 'result_value_reported: 1.6', 'result_unit_reported: NULL')
('study_time_collected: 56 Days', 'name_reported: MCHC', 'result_value_reported: 35', 'result_unit_reported: %')
('study_time_collected: 56 Days', 'name_reported: EOSINOPHILS', 'result_value_reported: 1.7', 'result_unit_reported: %')
('study_time_collected: 56 Days', 'name_reported: HEMATOCRIT', 'result_value_reported: 44', 'result_unit_reported: %')
('study_time_collected: 56 Days', 'name_reported: MCV', 'result_value_reported: 86', 'result_unit_reported: um*3')
('study_time_collected: 56 Days', 'name_reported: WBC', 'result_value_reported: 6.8', 'result_unit_reported: 10*3 cells/uL')
('study_time_collected: 56 Days', 'name_reported: MONOCYTES', 'result_value_reported: 4.3', 'result_unit_reported: %')
('study_time_collected: 56 Days', 'name_reported: LYMPHOCYTES', 'result_value_reported: 32.8', 'result_unit_reported: %')
('study_time_collected: 56 Days', 'name_reported: HEMOGLOBIN', 'result_value_reported: 15.3', 'result_unit_reported: g/dL')
('study_time_collected: 87 Days', 'name_reported: WBC', 'result_value_reported: 6.9', 'result_unit_reported: 10*3 cells/uL')
('study_time_collected: 87 Days', 'name_reported: RBC', 'result_value_reported: 5.21', 'result_unit_reported: 10*6 cells/uL')
('study_time_collected: 87 Days', 'name_reported: MCH', 'result_value_reported: 29', 'result_unit_reported: %')

('study_time_collected: 87 Days', 'name_reported: EOSINOPHILS', 'result_value_reported: 1.5', 'result_unit_reported: %')
('study_time_collected: 87 Days', 'name_reported: LYMPHOCYTES', 'result_value_reported: 39.1', 'result_unit_reported: %')
('study_time_collected: 87 Days', 'name_reported: MCV', 'result_value_reported: 84', 'result_unit_reported: um*3')
('study_time_collected: 87 Days', 'name_reported: SEGMENTED NEUTROPHILS', 'result_value_reported: 51.6', 'result_unit_reported: %')
('study_time_collected: 87 Days', 'name_reported: LARGE UNCLASSIFIED CELLS', 'result_value_reported: 1.8', 'result_unit_reported: NULL')
('study_time_collected: 87 Days', 'name_reported: HEMOGLOBIN', 'result_value_reported: 14.9', 'result_unit_reported: g/dL')
('study_time_collected: 87 Days', 'name_reported: PLATELET COUNT', 'result_value_reported: 238', 'result_unit_reported: 10*3 cells/uL')
('study_time_collected: 87 Days', 'name_reported: HEMATOCRIT', 'result_value_reported: 44', 'result_unit_reported: %')
('study_time_collected: 87 Days', 'name_reported: BASOPHILS', 'result_value_reported: 0.5', 'result_unit_reported: %')
('study_time_collected: 87 Days', 'name_reported: MCHC', 'result_value_reported: 34', 'result_unit_reported: %')
('study_time_collected: 87 Days', 'name_reported: MONOCYTES', 'result_value_reported: 5.5', 'result_unit_reported: %')

Assay Measurements
('study_time_collected: 100', 'analyte: IgG-a Ragweed', 'value_reported: 2307', 'unit_reported: ng/ml')
('study_time_collected: 100', 'analyte: IgE-a Amb a', 'value_reported: 2631.5', 'unit_reported: U/ml')
('study_time_collected: 100', 'analyte: IgG-a Amb a', 'value_reported: 4979.2', 'unit_reported: U/ml')
('study_time_collected: 100', 'analyte: Free IgE Concentration', 'value_reported: 21.967', 'unit_reported: ng/ml')
('study_time_collected: 100', 'analyte: IgE-a Ragweed', 'value_reported: 47.9', 'unit_reported: kIUa/ml')
('study_time_collected: 66', 'analyte: Free IgE Concentration', 'value_reported: 15.556', 'unit_reported: ng/ml')
('study_time_collected: 66', 'analyte: IgG-a Amb a', 'value_reported: 390.3', 'unit_reported: U/ml')
('study_time_collected: 66', 'analyte: IgE-a Ragweed', 'value_reported: 32.4', 'unit_reported: kIUa/ml')
('study_time_collected: 66', 'analyte: IgE-a Amb a', 'value_reported: 670.9', 'unit_reported: U/ml')
('study_time_collected: 66', 'analyte: IgG-a Ragweed', 'value_reported: 100', 'unit_reported: ng/ml')
('study_time_collected: 73', 'analyte: Free IgE Concentration', 'value_reported: 19.712', 'unit_reported: ng/ml')

('study_time_collected: 73', 'analyte: IgE-a Amb a', 'value_reported: 1005.5', 'unit_reported: U/ml')
('study_time_collected: 73', 'analyte: IgG-a Amb a', 'value_reported: 659.7', 'unit_reported: U/ml')
('study_time_collected: 73', 'analyte: IgE-a Ragweed', 'value_reported: 41', 'unit_reported: kIUa/ml')
('study_time_collected: 73', 'analyte: IgG-a Ragweed', 'value_reported: 290', 'unit_reported: ng/ml')

Adverse Reactions

Parotid duct cyst
Chest tightness
Injection site reaction NOS
Cough
Sinus congestion
Injection site pruritus
Dizziness
Rash pruritic
Sinus headache
Injection site pruritus
Blood creatine phosphokinase increased
Blood pressure decreased
Bursitis
Lower respiratory tract infection NOS
Bronchitis NOS
Nasopharyngitis
Sinusitis NOS
Throat tightness
Injection site pruritus
Dermatitis atopic

Supplementary Note 3

Subject: SUB73491
verification code: 741be0d4c0
Meta Information
race: Black or African American
gender: Male
age_reported: 28
arm_accession: ARM3
Blood Tests
('study_time_collected: -20 Days', 'name_reported: MCV', '90', 'um*3')
('study_time_collected: -20 Days', 'name_reported: WBC', '7.2', '10*3 cells/uL')
('study_time_collected: -20 Days', 'name_reported: MONOCYTES', '5.4', '%')
('study_time_collected: -20 Days', 'name_reported: MCHC', '34', '%')
('study_time_collected: -20 Days', 'name_reported: RBC', '4.71', '10*6 cells/uL')
('study_time_collected: -20 Days', 'name_reported: BASOPHILS', '0.2', '%')
('study_time_collected: -20 Days', 'name_reported: MCH', '30', '%')
('study_time_collected: -20 Days', 'name_reported: Total IgE', '34', 'unknown')
('study_time_collected: -20 Days', 'name_reported: HEMOGLOBIN', '14.1', 'g/dL')
('study_time_collected: -20 Days', 'name_reported: SEGMENTED NEUTROPHILS', '52.7', '%')
('study_time_collected: -20 Days', 'name_reported: LYMPHOCYTES', '37.1', '%')
('study_time_collected: -20 Days', 'name_reported: HEMATOCRIT', '42', '%')
('study_time_collected: -20 Days', 'name_reported: LARGE UNCLASSIFIED CELLS', '3.4', 'NULL')
('study_time_collected: -20 Days', 'name_reported: EOSINOPHILS', '1.7', '%')
('study_time_collected: -20 Days', 'name_reported: PLATELET COUNT', '194', '10*3 cells/uL')
('study_time_collected: 0 Days', 'name_reported: EOSINOPHILS', '4.6', '%')
('study_time_collected: 0 Days', 'name_reported: RBC', '4.45', '10*6 cells/uL')
('study_time_collected: 0 Days', 'name_reported: PLATELET COUNT', '223', '10*3 cells/uL')
('study_time_collected: 0 Days', 'name_reported: HEMATOCRIT', '41', '%')
('study_time_collected: 0 Days', 'name_reported: MCV', '92', 'um*3')
('study_time_collected: 0 Days', 'name_reported: MCHC', '33', '%')
('study_time_collected: 0 Days', 'name_reported: BASOPHILS', '0.4', '%')
('study_time_collected: 0 Days', 'name_reported: HEMOGLOBIN', '13.5', 'g/dL')
('study_time_collected: 0 Days', 'name_reported: MCH', '30', '%')
('study_time_collected: 0 Days', 'name_reported: MONOCYTES', '6.6', '%')
('study_time_collected: 0 Days', 'name_reported: LYMPHOCYTES', '41.2', '%')
('study_time_collected: 0 Days', 'name_reported: WBC', '5.9', '10*3 cells/uL')
('study_time_collected: 0 Days', 'name_reported: BANDS', '0', '%')
('study_time_collected: 0 Days', 'name_reported: SEGMENTED NEUTROPHILS', '44.4', '%')
('study_time_collected: 0 Days', 'name_reported: LARGE UNCLASSIFIED CELLS', '2.8', 'NULL')

('study_time_collected: 119 Days', 'name_reported: LYMPHOCYTES', '38.1', '%')
('study_time_collected: 119 Days', 'name_reported: WBC', '5.4', '10*3 cells/uL')
('study_time_collected: 119 Days', 'name_reported: RBC', '4.55', '10*6 cells/uL')
('study_time_collected: 119 Days', 'name_reported: MCHC', '34', '%')
('study_time_collected: 119 Days', 'name_reported: MCV', '88', 'um*3')
('study_time_collected: 119 Days', 'name_reported: PLATELET COUNT', '189', '10*3 cells/uL')
('study_time_collected: 119 Days', 'name_reported: SEGMENTED NEUTROPHILS', '50.2', '%')
('study_time_collected: 119 Days', 'name_reported: MONOCYTES', '6.2', '%')
('study_time_collected: 119 Days', 'name_reported: HEMOGLOBIN', '13.7', 'g/dL')
('study_time_collected: 119 Days', 'name_reported: EOSINOPHILS', '2.4', '%')
('study_time_collected: 119 Days', 'name_reported: LARGE UNCLASSIFIED CELLS', '2.6', 'NULL')
('study_time_collected: 119 Days', 'name_reported: MCH', '30', '%')
('study_time_collected: 119 Days', 'name_reported: BASOPHILS', '0.5', '%')
('study_time_collected: 119 Days', 'name_reported: HEMATOCRIT', '40', '%')
('study_time_collected: 147 Days', 'name_reported: MCH', '30', '%')
('study_time_collected: 147 Days', 'name_reported: HEMOGLOBIN', '13.7', 'g/dL')
('study_time_collected: 147 Days', 'name_reported: HEMATOCRIT', '41', '%')
('study_time_collected: 147 Days', 'name_reported: MCV', '90', 'um*3')
('study_time_collected: 147 Days', 'name_reported: EOSINOPHILS', '2.6', '%')
('study_time_collected: 147 Days', 'name_reported: LARGE UNCLASSIFIED CELLS', '2.4', 'NULL')
('study_time_collected: 147 Days', 'name_reported: SEGMENTED NEUTROPHILS', '54.2', '%')
('study_time_collected: 147 Days', 'name_reported: MONOCYTES', '6.5', '%')
('study_time_collected: 147 Days', 'name_reported: RBC', '4.51', '10*6 cells/uL')
('study_time_collected: 147 Days', 'name_reported: PLATELET COUNT', '203', '10*3 cells/uL')
('study_time_collected: 147 Days', 'name_reported: WBC', '6.9', '10*3 cells/uL')
('study_time_collected: 147 Days', 'name_reported: LYMPHOCYTES', '34', '%')
('study_time_collected: 147 Days', 'name_reported: MCHC', '34', '%')
('study_time_collected: 147 Days', 'name_reported: BASOPHILS', '0.3', '%')
('study_time_collected: 160 Days', 'name_reported: RBC', '4.29', '10*6 cells/uL')
('study_time_collected: 160 Days', 'name_reported: MONOCYTES', '7.3', '%')
('study_time_collected: 160 Days', 'name_reported: HEMATOCRIT', '39', '%')
('study_time_collected: 160 Days', 'name_reported: MCHC', '34', '%')
('study_time_collected: 160 Days', 'name_reported: LYMPHOCYTES', '37', '%')
('study_time_collected: 160 Days', 'name_reported: MCH', '31', '%')
('study_time_collected: 160 Days', 'name_reported: HEMOGLOBIN', '13.3', 'g/dL')
('study_time_collected: 160 Days', 'name_reported: BASOPHILS', '0.3', '%')
('study_time_collected: 160 Days', 'name_reported: EOSINOPHILS', '2.8', '%')
('study_time_collected: 160 Days', 'name_reported: LARGE UNCLASSIFIED CELLS', '2.9', 'NULL')
('study_time_collected: 160 Days', 'name_reported: WBC', '4.7', '10*3 cells/uL')

('study_time_collected: 160 Days', 'name_reported: PLATELET COUNT', '198', '10*3 cells/uL')
('study_time_collected: 160 Days', 'name_reported: MCV', '90', 'um*3')
('study_time_collected: 160 Days', 'name_reported: SEGMENTED NEUTROPHILS', '49.7', '%')
('study_time_collected: 28 Days', 'name_reported: WBC', '6.2', '10*3 cells/uL')
('study_time_collected: 28 Days', 'name_reported: BASOPHILS', '0', '%')
('study_time_collected: 28 Days', 'name_reported: BANDS', '4', '%')
('study_time_collected: 28 Days', 'name_reported: PLATELET COUNT', '235', '10*3 cells/uL')
('study_time_collected: 28 Days', 'name_reported: LYMPHOCYTES', '49', '%')
('study_time_collected: 28 Days', 'name_reported: MCHC', '33', '%')
('study_time_collected: 28 Days', 'name_reported: EOSINOPHILS', '0', '%')
('study_time_collected: 28 Days', 'name_reported: SEGMENTED NEUTROPHILS', '43', '%')
('study_time_collected: 28 Days', 'name_reported: HEMATOCRIT', '43', '%')
('study_time_collected: 28 Days', 'name_reported: MCV', '91', 'um*3')
('study_time_collected: 28 Days', 'name_reported: RBC', '4.68', '10*6 cells/uL')
('study_time_collected: 28 Days', 'name_reported: MCH', '30', '%')
('study_time_collected: 28 Days', 'name_reported: HEMOGLOBIN', '14.2', 'g/dL')
('study_time_collected: 28 Days', 'name_reported: MONOCYTES', '4', '%')
('study_time_collected: 369 Days', 'name_reported: BASOPHILS', '0.3', '%')
('study_time_collected: 369 Days', 'name_reported: SEGMENTED NEUTROPHILS', '47.7', '%')
('study_time_collected: 369 Days', 'name_reported: LYMPHOCYTES', '39.5', '%')
('study_time_collected: 369 Days', 'name_reported: EOSINOPHILS', '2.3', '%')
('study_time_collected: 369 Days', 'name_reported: HEMOGLOBIN', '14.4', 'g/dL')
('study_time_collected: 369 Days', 'name_reported: LARGE UNCLASSIFIED CELLS', '2.8', 'NULL')
('study_time_collected: 369 Days', 'name_reported: HEMATOCRIT', '42', '%')
('study_time_collected: 369 Days', 'name_reported: MCH', '31', '%')
('study_time_collected: 369 Days', 'name_reported: MONOCYTES', '7.4', '%')
('study_time_collected: 369 Days', 'name_reported: MCV', '91', 'um*3')
('study_time_collected: 369 Days', 'name_reported: MCHC', '34', '%')
('study_time_collected: 369 Days', 'name_reported: RBC', '4.62', '10*6 cells/uL')
('study_time_collected: 369 Days', 'name_reported: WBC', '5.2', '10*3 cells/uL')
('study_time_collected: 369 Days', 'name_reported: PLATELET COUNT', '179', '10*3 cells/uL')
('study_time_collected: 56 Days', 'name_reported: BANDS', '1', '%')
('study_time_collected: 56 Days', 'name_reported: MCV', '90', 'um*3')
('study_time_collected: 56 Days', 'name_reported: HEMATOCRIT', '43', '%')
('study_time_collected: 56 Days', 'name_reported: MONOCYTES', '9', '%')
('study_time_collected: 56 Days', 'name_reported: HEMOGLOBIN', '14.5', 'g/dL')
('study_time_collected: 56 Days', 'name_reported: BASOPHILS', '0', '%')
('study_time_collected: 56 Days', 'name_reported: MCHC', '34', '%')
('study_time_collected: 56 Days', 'name_reported: RBC', '4.74', '10*6 cells/uL')

('study_time_collected: 56 Days', 'name_reported: MCH', '31', '%')
('study_time_collected: 56 Days', 'name_reported: LYMPHOCYTES', '51', '%')
('study_time_collected: 56 Days', 'name_reported: PLATELET COUNT', '221', '10*3 cells/uL')
('study_time_collected: 56 Days', 'name_reported: WBC', '6.7', '10*3 cells/uL')
('study_time_collected: 56 Days', 'name_reported: SEGMENTED NEUTROPHILS', '39', '%')
('study_time_collected: 56 Days', 'name_reported: EOSINOPHILS', '0', '%')
('study_time_collected: 91 Days', 'name_reported: BASOPHILS', '0.3', '%')
('study_time_collected: 91 Days', 'name_reported: LYMPHOCYTES', '30.3', '%')
('study_time_collected: 91 Days', 'name_reported: PLATELET COUNT', '199', '10*3 cells/uL')
('study_time_collected: 91 Days', 'name_reported: MONOCYTES', '5.5', '%')
('study_time_collected: 91 Days', 'name_reported: EOSINOPHILS', '1.9', '%')
('study_time_collected: 91 Days', 'name_reported: SEGMENTED NEUTROPHILS', '59.6', '%')
('study_time_collected: 91 Days', 'name_reported: MCHC', '35', '%')
('study_time_collected: 91 Days', 'name_reported: HEMOGLOBIN', '13.8', 'g/dL')
('study_time_collected: 91 Days', 'name_reported: LARGE UNCLASSIFIED CELLS', '2.4', 'NULL')
('study_time_collected: 91 Days', 'name_reported: RBC', '4.53', '10*6 cells/uL')
('study_time_collected: 91 Days', 'name_reported: MCH', '30', '%')
('study_time_collected: 91 Days', 'name_reported: MCV', '88', 'um*3')
('study_time_collected: 91 Days', 'name_reported: HEMATOCRIT', '40', '%')
('study_time_collected: 91 Days', 'name_reported: WBC', '6.8', '10*3 cells/uL')

Assay Measurements

('study_time_collected: -8', 'analyte: IgE-a Amb a', '28.3', 'U/ml')
('study_time_collected: -8', 'analyte: IgG-a Amb a', '43', 'U/ml')
('study_time_collected: -8', 'analyte: IgE-a Ragweed', '1.71', 'kIUa/ml')
('study_time_collected: -8', 'analyte: Free IgE Concentration', '89.92', 'ng/ml')
('study_time_collected: -8', 'analyte: IgG-a Ragweed', '100', 'ng/ml')
('study_time_collected: 105', 'analyte: IgG-a Amb a', '511.5', 'U/ml')
('study_time_collected: 105', 'analyte: IgE-a Amb a', '3340', 'U/ml')
('study_time_collected: 105', 'analyte: IgE-a Ragweed', '71.1', 'kIUa/ml')
('study_time_collected: 105', 'analyte: Free IgE Concentration', '17.376', 'ng/ml')
('study_time_collected: 105', 'analyte: IgG-a Ragweed', '385', 'ng/ml')
('study_time_collected: 202', 'analyte: Free IgE Concentration', '34.303', 'ng/ml')
('study_time_collected: 202', 'analyte: IgE-a Ragweed', '45.6', 'kIUa/ml')
('study_time_collected: 202', 'analyte: IgG-a Ragweed', '377', 'ng/ml')
('study_time_collected: 202', 'analyte: IgG-a Amb a', '398', 'U/ml')
('study_time_collected: 202', 'analyte: IgE-a Amb a', '2195', 'U/ml')

Adverse Reactions

None

Supplementary Note 4

Subject: SUB73511
verification code: d3feabcf82
Meta Information
race: White
gender: Male
age_reported: 45
arm_accession: ARM4
Blood Tests
('study_time_collected: -15 Days', 'name_reported: RBC', '5.45', '10*6 cells/uL')
('study_time_collected: -15 Days', 'name_reported: HEMATOCRIT', '47', '%')
('study_time_collected: -15 Days', 'name_reported: PLATELET COUNT', '271', '10*3 cells/uL')
('study_time_collected: -15 Days', 'name_reported: WBC', '6.7', '10*3 cells/uL')
('study_time_collected: -15 Days', 'name_reported: HEMOGLOBIN', '15.8', 'g/dL')
('study_time_collected: -15 Days', 'name_reported: MCV', '86', 'um*3')
('study_time_collected: -15 Days', 'name_reported: Total IgE', '58', 'unknown')
('study_time_collected: -15 Days', 'name_reported: MCHC', '34', '%')
('study_time_collected: -15 Days', 'name_reported: MCH', '29', '%')
('study_time_collected: 0 Days', 'name_reported: LARGE UNCLASSIFIED CELLS', '2.2', 'NULL')
('study_time_collected: 0 Days', 'name_reported: MONOCYTES', '4.5', '%')
('study_time_collected: 0 Days', 'name_reported: HEMOGLOBIN', '14.8', 'g/dL')
('study_time_collected: 0 Days', 'name_reported: MCV', '87', 'um*3')
('study_time_collected: 0 Days', 'name_reported: EOSINOPHILS', '1.9', '%')
('study_time_collected: 0 Days', 'name_reported: SEGMENTED NEUTROPHILS', '56.3', '%')
('study_time_collected: 0 Days', 'name_reported: HEMATOCRIT', '44', '%')
('study_time_collected: 0 Days', 'name_reported: WBC', '6.8', '10*3 cells/uL')
('study_time_collected: 0 Days', 'name_reported: BASOPHILS', '0.6', '%')
('study_time_collected: 0 Days', 'name_reported: MCH', '29', '%')
('study_time_collected: 0 Days', 'name_reported: LYMPHOCYTES', '34.5', '%')
('study_time_collected: 0 Days', 'name_reported: MCHC', '34', '%')
('study_time_collected: 0 Days', 'name_reported: RBC', '5.09', '10*6 cells/uL')
('study_time_collected: 0 Days', 'name_reported: PLATELET COUNT', '295', '10*3 cells/uL')
('study_time_collected: 114 Days', 'name_reported: LARGE UNCLASSIFIED CELLS', '2.4', 'NULL')
('study_time_collected: 114 Days', 'name_reported: PLATELET COUNT', '278', '10*3 cells/uL')
('study_time_collected: 114 Days', 'name_reported: HEMATOCRIT', '46', '%')
('study_time_collected: 114 Days', 'name_reported: BASOPHILS', '0.6', '%')
('study_time_collected: 114 Days', 'name_reported: SEGMENTED NEUTROPHILS', '57.3', '%')
('study_time_collected: 114 Days', 'name_reported: EOSINOPHILS', '2', '%')

('study_time_collected: 114 Days', 'name_reported: LYMPHOCYTES', '32.5', '%')
('study_time_collected: 114 Days', 'name_reported: MCH', '29', '%')
('study_time_collected: 114 Days', 'name_reported: MCHC', '34', '%')
('study_time_collected: 114 Days', 'name_reported: WBC', '7.3', '10*3 cells/uL')
('study_time_collected: 114 Days', 'name_reported: MONOCYTES', '5.2', '%')
('study_time_collected: 114 Days', 'name_reported: HEMOGLOBIN', '15.5', 'g/dL')
('study_time_collected: 114 Days', 'name_reported: RBC', '5.45', '10*6 cells/uL')
('study_time_collected: 114 Days', 'name_reported: MCV', '85', 'um*3')
('study_time_collected: 140 Days', 'name_reported: MCV', '84', 'um*3')
('study_time_collected: 140 Days', 'name_reported: HEMOGLOBIN', '15.1', 'g/dL')
('study_time_collected: 140 Days', 'name_reported: LARGE UNCLASSIFIED CELLS', '0.9', 'NULL')
('study_time_collected: 140 Days', 'name_reported: BASOPHILS', '0.4', '%')
('study_time_collected: 140 Days', 'name_reported: MONOCYTES', '3.5', '%')
('study_time_collected: 140 Days', 'name_reported: WBC', '12.7', '10*3 cells/uL')
('study_time_collected: 140 Days', 'name_reported: MCH', '29', '%')
('study_time_collected: 140 Days', 'name_reported: EOSINOPHILS', '0.5', '%')
('study_time_collected: 140 Days', 'name_reported: MCHC', '34', '%')
('study_time_collected: 140 Days', 'name_reported: SEGMENTED NEUTROPHILS', '77.8', '%')
('study_time_collected: 140 Days', 'name_reported: HEMATOCRIT', '44', '%')
('study_time_collected: 140 Days', 'name_reported: PLATELET COUNT', '275', '10*3 cells/uL')
('study_time_collected: 140 Days', 'name_reported: LYMPHOCYTES', '16.9', '%')
('study_time_collected: 140 Days', 'name_reported: RBC', '5.26', '10*6 cells/uL')
('study_time_collected: 155 Days', 'name_reported: MCV', '84', 'um*3')
('study_time_collected: 155 Days', 'name_reported: MONOCYTES', '4.8', '%')
('study_time_collected: 155 Days', 'name_reported: HEMATOCRIT', '44', '%')
('study_time_collected: 155 Days', 'name_reported: PLATELET COUNT', '286', '10*3 cells/uL')
('study_time_collected: 155 Days', 'name_reported: LYMPHOCYTES', '33.3', '%')
('study_time_collected: 155 Days', 'name_reported: MCH', '30', '%')
('study_time_collected: 155 Days', 'name_reported: LARGE UNCLASSIFIED CELLS', '1.7', 'NULL')
('study_time_collected: 155 Days', 'name_reported: HEMOGLOBIN', '15.7', 'g/dL')
('study_time_collected: 155 Days', 'name_reported: BASOPHILS', '0.9', '%')
('study_time_collected: 155 Days', 'name_reported: WBC', '7.2', '10*3 cells/uL')
('study_time_collected: 155 Days', 'name_reported: EOSINOPHILS', '2.4', '%')
('study_time_collected: 155 Days', 'name_reported: SEGMENTED NEUTROPHILS', '56.9', '%')
('study_time_collected: 155 Days', 'name_reported: RBC', '5.21', '10*6 cells/uL')
('study_time_collected: 155 Days', 'name_reported: MCHC', '36', '%')
('study_time_collected: 28 Days', 'name_reported: SEGMENTED NEUTROPHILS', '64.3', '%')
('study_time_collected: 28 Days', 'name_reported: HEMOGLOBIN', '15.8', 'g/dL')

('study_time_collected: 28 Days', 'name_reported: LARGE UNCLASSIFIED CELLS', '1.7', 'NULL')
('study_time_collected: 28 Days', 'name_reported: RBC', '5.44', '10*6 cells/uL')
('study_time_collected: 28 Days', 'name_reported: MCV', '87', 'um*3')
('study_time_collected: 28 Days', 'name_reported: BASOPHILS', '0.7', '%')
('study_time_collected: 28 Days', 'name_reported: MCHC', '33', '%')
('study_time_collected: 28 Days', 'name_reported: PLATELET COUNT', '267', '10*3 cells/uL')
('study_time_collected: 28 Days', 'name_reported: WBC', '6.5', '10*3 cells/uL')
('study_time_collected: 28 Days', 'name_reported: EOSINOPHILS', '1.3', '%')
('study_time_collected: 28 Days', 'name_reported: LYMPHOCYTES', '29.2', '%')
('study_time_collected: 28 Days', 'name_reported: HEMATOCRIT', '47', '%')
('study_time_collected: 28 Days', 'name_reported: MONOCYTES', '3', '%')
('study_time_collected: 28 Days', 'name_reported: MCH', '29', '%')
('study_time_collected: 365 Days', 'name_reported: MCHC', '33', '%')
('study_time_collected: 365 Days', 'name_reported: HEMOGLOBIN', '15.1', 'g/dL')
('study_time_collected: 365 Days', 'name_reported: SEGMENTED NEUTROPHILS', '52.8', '%')
('study_time_collected: 365 Days', 'name_reported: LARGE UNCLASSIFIED CELLS', '1.7', 'NULL')
('study_time_collected: 365 Days', 'name_reported: MCV', '87', 'um*3')
('study_time_collected: 365 Days', 'name_reported: MONOCYTES', '6.2', '%')
('study_time_collected: 365 Days', 'name_reported: RBC', '5.19', '10*6 cells/uL')
('study_time_collected: 365 Days', 'name_reported: HEMATOCRIT', '45', '%')
('study_time_collected: 365 Days', 'name_reported: EOSINOPHILS', '2.7', '%')
('study_time_collected: 365 Days', 'name_reported: LYMPHOCYTES', '36.3', '%')
('study_time_collected: 365 Days', 'name_reported: MCH', '29', '%')
('study_time_collected: 365 Days', 'name_reported: WBC', '5.9', '10*3 cells/uL')
('study_time_collected: 365 Days', 'name_reported: BASOPHILS', '0.3', '%')
('study_time_collected: 365 Days', 'name_reported: PLATELET COUNT', '252', '10*3 cells/uL')
('study_time_collected: 56 Days', 'name_reported: RBC', '5.17', '10*6 cells/uL')
('study_time_collected: 56 Days', 'name_reported: SEGMENTED NEUTROPHILS', '58.9', '%')
('study_time_collected: 56 Days', 'name_reported: PLATELET COUNT', '270', '10*3 cells/uL')
('study_time_collected: 56 Days', 'name_reported: MCH', '30', '%')
('study_time_collected: 56 Days', 'name_reported: BASOPHILS', '0.7', '%')
('study_time_collected: 56 Days', 'name_reported: LARGE UNCLASSIFIED CELLS', '1.6', 'NULL')
('study_time_collected: 56 Days', 'name_reported: MCHC', '35', '%')
('study_time_collected: 56 Days', 'name_reported: EOSINOPHILS', '1.7', '%')
('study_time_collected: 56 Days', 'name_reported: HEMATOCRIT', '44', '%')
('study_time_collected: 56 Days', 'name_reported: MCV', '86', 'um*3')
('study_time_collected: 56 Days', 'name_reported: WBC', '6.8', '10*3 cells/uL')
('study_time_collected: 56 Days', 'name_reported: MONOCYTES', '4.3', '%')

('study_time_collected: 56 Days', 'name_reported: LYMPHOCYTES', '32.8', '%')
('study_time_collected: 56 Days', 'name_reported: HEMOGLOBIN', '15.3', 'g/dL')
('study_time_collected: 87 Days', 'name_reported: WBC', '6.9', '10*3 cells/uL')
('study_time_collected: 87 Days', 'name_reported: RBC', '5.21', '10*6 cells/uL')
('study_time_collected: 87 Days', 'name_reported: MCH', '29', '%')
('study_time_collected: 87 Days', 'name_reported: EOSINOPHILS', '1.5', '%')
('study_time_collected: 87 Days', 'name_reported: LYMPHOCYTES', '39.1', '%')
('study_time_collected: 87 Days', 'name_reported: MCV', '84', 'um*3')
('study_time_collected: 87 Days', 'name_reported: SEGMENTED NEUTROPHILS', '51.6', '%')
('study_time_collected: 87 Days', 'name_reported: LARGE UNCLASSIFIED CELLS', '1.8', 'NULL')
('study_time_collected: 87 Days', 'name_reported: HEMOGLOBIN', '14.9', 'g/dL')
('study_time_collected: 87 Days', 'name_reported: PLATELET COUNT', '238', '10*3 cells/uL')
('study_time_collected: 87 Days', 'name_reported: HEMATOCRIT', '44', '%')
('study_time_collected: 87 Days', 'name_reported: BASOPHILS', '0.5', '%')
('study_time_collected: 87 Days', 'name_reported: MCHC', '34', '%')
('study_time_collected: 87 Days', 'name_reported: MONOCYTES', '5.5', '%')

Assay Measurements
('study_time_collected: 100', 'analyte: IgG-a Ragweed', '2307', 'ng/ml')
('study_time_collected: 100', 'analyte: IgE-a Amb a', '2631.5', 'U/ml')
('study_time_collected: 100', 'analyte: IgG-a Amb a', '4979.2', 'U/ml')
('study_time_collected: 100', 'analyte: Free IgE Concentration', '21.967', 'ng/ml')
('study_time_collected: 100', 'analyte: IgE-a Ragweed', '47.9', 'kIUa/ml')
('study_time_collected: 66', 'analyte: Free IgE Concentration', '15.556', 'ng/ml')
('study_time_collected: 66', 'analyte: IgG-a Amb a', '390.3', 'U/ml')
('study_time_collected: 66', 'analyte: IgE-a Ragweed', '32.4', 'kIUa/ml')
('study_time_collected: 66', 'analyte: IgE-a Amb a', '670.9', 'U/ml')
('study_time_collected: 66', 'analyte: IgG-a Ragweed', '100', 'ng/ml')
('study_time_collected: 73', 'analyte: Free IgE Concentration', '19.712', 'ng/ml')
('study_time_collected: 73', 'analyte: IgE-a Amb a', '1005.5', 'U/ml')
('study_time_collected: 73', 'analyte: IgG-a Amb a', '659.7', 'U/ml')
('study_time_collected: 73', 'analyte: IgE-a Ragweed', '41', 'kIUa/ml')
('study_time_collected: 73', 'analyte: IgG-a Ragweed', '290', 'ng/ml')

Adverse Reactions
None
